# Gating Properties of Mutant Sodium Channels and Responses to Sodium Current Inhibitors Predict Mexiletine-Sensitive Mutations of Long QT Syndrome 3

**DOI:** 10.3389/fphar.2020.01182

**Published:** 2020-08-04

**Authors:** Gang Li, Ryan L. Woltz, Cheng-yu Wang, Lu Ren, Pei-xin He, Shan-dong Yu, Xue-qin Liu, Vladimir Yarov-Yarovoy, Dan Hu, Nipavan Chiamvimonvat, Lin Wu

**Affiliations:** ^1^ Department of Cardiology, Peking University First Hospital, Beijing, China; ^2^ Division of Cardiovascular Medicine, Department of Internal Medicine, School of Medicine, University of California, Davis, Davis, CA, United States; ^3^ Department of Pediatrics, Peking University First Hospital, Beijing, China; ^4^ Department of Physiology and Membrane Biology, School of Medicine, University of California, Davis, Davis, CA, United States; ^5^ Department of Cardiology and Cardiovascular Research Institute, Renmin Hospital of Wuhan University and Hubei Key Laboratory of Cardiology, Wuhan, China; ^6^ Department of Veterans Affairs, Northern California Health Care System, Mather, CA, United States; ^7^ Key Laboratory of Medical Electrophysiology, Institute of Cardiovascular Research, Southwest Medical University, Luzhou, China

**Keywords:** LQT3, torsades de pointes, gene mutation, late sodium current, mexiletine

## Abstract

**Background:**

Long QT syndrome 3 (LQT3) is caused by *SCN5A* mutations. Late sodium current (late *I*_Na_) inhibitors are current-specific to treat patients with LQT3, but the mechanisms underlying mexiletine (MEX) -sensitive (N1325S and R1623Q) and -insensitive (M1652R) mutations remains to be elucidated.

**Methods:**

LQT3 patients with causative mutations were treated with oral MEX following i.v. lidocaine. Whole-cell patch-clamp techniques and molecular remodeling were used to determine the mechanisms underlying the sensitivity to MEX.

**Results:**

Intravenous administration of lidocaine followed by MEX orally in LQT patients with N1325S and R1623Q sodium channel mutation shortened QTc interval, abolished arrhythmias, and completely normalized the ECG. In HEK293 cells, the steady-state inactivation curves of the M1652R channels were rightward shifted by 5.6 mV relative to the WT channel. In contrast, the R1623Q mutation caused a leftward shift of the steady-state inactivation curve by 15.2 mV compared with WT channel, and N1325S mutation did not affect steady-state inactivation (n = 5–13, *P* < 0.05). The extent of the window current was expanded in all three mutant channels compared with WT. All three mutations increased late *I*_Na_ with the greatest amplitude in the M1652R channel (n = 9–15, *P* < 0.05). MEX caused a hyperpolarizing shift of the steady-state inactivation and delayed the recovery of all three mutant channels. Furthermore, it suppressed late *I*_Na_ in N1325S and R1623Q to a greater extent compared to that of M1652R mutant channel. Mutations altered the sensitivity of Na_v_1.5 to MEX through allosteric mechanisms by changing the conformation of Na_v_1.5 to become more or less favorable for MEX binding. Late *I*_Na_ inhibitors suppressed late *I*_Na_ in N1325S and R1623Q to a greater extent than that in the M1652R mutation (n = 4–7, *P* < 0.05).

**Conclusion:**

The N1325S, R1623Q, and M1652R mutations are associated with a variable augmentation of late *I*_Na_, which was reversed by MEX. M1652R mutation changes the conformation of Na_v_1.5 that disrupt the inactivation of channel affecting MEX binding, corresponding to the poor response to MEX. The lidocaine test, molecular modeling, and drugs screening in cells expressing mutant channels are useful for predicting the effectiveness of late *I*_Na_ inhibitors.

## Introduction

Congenital long QT syndrome (LQTS) is a genetic disorder caused by ion channel mutations disrupting the electrical activity of the heart with a prevalence of approximately 1:2000 (Garcia-Elias and Benito, 2018) in apparently healthy live births. Patients with the mutation(s) may present with QT interval prolongation in ECG recordings with episodes of polymorphic ventricular tachycardia, specifically torsade de pointes (TdP), resulting in syncope, cardiac arrest, and sudden cardiac death. To date, at least 15 genes have been identified and are associated with LQTS. LQTS types 1 and 2 caused by K^+^ channel mutations account for approximately two-thirds of genetically confirmed LQTS patients (Wilde et al., 2016; Bohnen et al., 2017; Wallace et al., 2019).

LQT3 is relatively rare accounting for 5 to 10% of LQTS patients but is more malignant than LQT1 or 2 with a 10-year survival rate of less than 50%. LQT3 is caused by gain-of-function mutations in the *SCN5A* gene encoding the α-subunit of Na_v_1.5 sodium channel, leading to abnormal Na^+^ channel activation and/or inactivation, resulting in a sustained or late inward sodium current (*I_Na_*). The increased late *I*_Na_ during the plateau phase of the cardiac action potential (AP) leads to prolonged AP duration (APD) and QT interval, increased propensities to pro-arrhythmic events including early (EAD) and delayed (DAD) afterdepolarizations, and enhanced transmural dispersions of repolarization and QT dispersion (George, 2005; Chadda et al., 2017; Yu et al., 2018). Furthermore, increased late *I*_Na_ is also documented in patients with LQT types 4, 9, 10, and 12. Both endogenous and enhanced late *I*_Na_ exhibit a frequency-dependent increase resulting in reverse rate dependence in APD (Wu et al., 2011; Yu et al., 2018). This property may explain why cardiac events of LQT3 patients often present at rest or during sleep, which is different from LQT1 and LQT2 patients whose arrhythmic events are associated with increased sympathetic activity and triggered by physical/emotional stress or environmental stimuli.

Anti-adrenergic therapies, including β-blockers and left cardiac sympathetic denervation, are recommended in guidelines to treat patients with LQTS, which are more effective in patients with LQT1 and LQT2 than LQT3. β-blockers are less effective and even potentially pro-arrhythmic associated with additional sudden death in case reports of patients with LQT3 after heart rate was slowed by drugs (Moss et al., 2000; Shimizu et al., 2000). ICD implantation is a great challenge in developing countries, especially in children. Drugs blocking late *I*_Na_ are considered as current-specific therapies for LQT3, because they directly reduce late *I*_Na_, shortens the QTc interval, and are antiarrhythmic, which are superior to β-blockers from the mechanistic point of view (Arbelo et al., 2016; Huang et al., 2020). Lidocaine (LID), mexiletine (MEX), ranolazine (RAN), and a novel late *I*_Na_ inhibitor eleclazine (ELE) have been reported to inhibit late *I*_Na_. However, patients with some mutations including M1652R mutation of LQT3 was reported to respond to MEX poorly (Ruan et al., 2007).

In this study, two patients with LQT3 associated with either N1325S or R1623Q mutation in *SCN5A* and responded well to LID and MEX treatment were evaluated. The electrophysiological properties and responses to MEX were compared in human embryonic kidney cells (HEK293) expressing N1325S, R1623Q, and M1652R mutant channels. A molecular model of Na_v_1.5 channel was also used to investigate the underlying mechanisms of their differences in sensitivity to MEX.

## Methods

### Clinical Evaluation

The research protocols described were approved by the Ethics Committee of Peking University First Hospital (Beijing, China). A written informed consent was obtained from the minor(s)’ legal guardian for the publication of any potentially identifiable image or data included in this article. LQTS was diagnosed based on standard clinical criteria and guidelines (Priori et al., 2013). Electrocardiogram (ECG) parameters were manually measured on standard 12-leads ECG recordings. The QT interval was measured in lead II or V5 on three consecutive beats and corrected by heart rate using Bazett’s formula. The LID infusion test was performed with a bolus intravenous injection of 1–2 mg/kg LID followed by an infusion of 50 μg/kg/min for 2 h. ECG recordings at baseline and at 5, 15, 30, 90, and 120 min after LID administration were obtained. Following LID infusion, MEX was administered orally 150 mg or 3–5 mg/Kg, three times daily.

### Site-Directed Mutagenesis and Transfection in HEK293 Cells

The mutations were engineered into wild-type (WT) *SCN5A* cDNA cloned in pcDNA3.1 by overlap extension with the following primer pairs: N1325S, forward: 5’-TGG TCA GTG CCC TGG TGG GCG CCA TC-3’, reverse: 5’-CAG GGC ACT GAC CAC CAC CCT CAT GC-3’; R1623Q, forward: 5’- TCT TCC AAG TCA TCC GCC TGG CCC G-3’, reverse: 5’- ATG ACT TGG AAG AGC GTC GGG GAG-3’; and M1652R, forward: 5’-CTC ATG AGG TCC CTG CCT GCC CTC TTC-3’, reverse: 5’- CAG GGA CCT CAT GAG GGC AAA GAG CAG C-3’. The mutations were confirmed by direct sequencing. Transient transfection was carried out using lipofectamine 3000 (Invitrogen) according to the manufacture’s instruction. A total of 2.0 μg of WT, N1325S, R1623Q, or M1652R *SCN5A* cDNA with *SCN1B* cDNA (encodes a β-1 subunit of the sodium channel) was used with equal amount of α and β subunits, and 0.2 μg of GFP plasmid as a report gene were transfected into HEK293 cells. GFP-positive cells were patch-clamped at least 24 h after transfection.

### *In Vitro* Electrophysiology Experiments

Whole-cell patch-clamp experiments were performed at room temperature (20–22°C) with an EPC-10 USB amplifier. Experiments were conducted with the following internal solution (in mM): 120 CsF, 10 CsCl, 10 NaCl, 10 EGTA, and 10 HEPES (pH 7.35) adjusted with CsOH (Wang et al., 1996). The bath solution contained (in mM): 137 NaCl, 4 KCl, 1.8 CaCl_2_, 1 MgCl_2_, 10 glucoses, 10 HEPES (pH 7.4) adjusted with CsOH. In experiments designed to measure parameters of peak *I*_Na_, external Na^+^ concentration was reduced to 60 mM with CsCl used as a Na^+^ substitute. Electrodes (3–5 MΩ) were pulled from 1.5 mm Sutter Instrument BF150-86-10 borosilicate glasses with filament using a PP-830 puller (NARISHIGE, Japan). After whole-cell configuration was achieved, only cells with seal resistance over 500 MΩ and access resistance less than 10 MΩ were recorded. Capacitance and series resistance compensation (80–90%) were carried out using analog techniques with patch-clamp amplifier. Pulse protocols are presented in each result figure. Currents were filtered at 2.9 kHz of Bessel filter and digitized at 10 kHz. Recordings were generally initiated at least 5 min after establishment of whole-cell configuration. Membrane potentials were not corrected for junction potentials that arose between the pipette and bath solution. All drugs were prepared as stock solutions in DMSO; concentrations of DMSO never exceeded 0.1% v/v in the final experimental solutions.

Late *I*_Na_ was measured as the tetrodotoxin (TTX; 30 μM)-sensitive current measured at 200 ms after a depolarization (a voltage step) from -90 mV to −20 mV was induced. Current densities (pA/pF) were obtained by dividing the peak or late *I*_Na_ by cell capacitance. Data for voltage dependence of activation and inactivation were fitted to a Boltzmann equation, y = 1/{1 + exp [(V_1/2_ - V_m_)/κ]}, in which y is the normalized current or conductance, V_1/2_ is the voltage at which half of the channels are activated or inactivated, V_m_ is the membrane potential, and κ is the slope factor. Data for the time course of recovery from inactivation were fitted with functions of two exponentials, P_2_/P_1_ = A_1_[1 - exp(-t/τ_fast_)] + A_2_[1 - exp(-t/τ_slow_)], where P_1_ and P_2_ are the peak sodium current of test pulse and pre-pulse and τ_fast_ and τ_slow_ are the fast and slow recovery time constants, respectively.

### Molecular Model of Na_v_1.5

A molecular model of Na_v_1.5 channel in a partially open and presumably inactivated state was previously generated using Rosetta structural modeling software and the cryoEM structure of the eeNav1.4-beta1 complex (Nguyen et al., 2019). The three LQTS3 mutations (N1325S, R1623Q, and M1652R) were generated in Na_v_1.5 channel using UCSF Chimera developed by the Resource for Biocomputing, Visualization, and Informatics at the University of California, San Francisco, with support from NIH P41-GM103311 (Pettersen et al., 2004). Molecular docking of MEX to wild-type Na_v_1.5 channel was performed using Rosetta Ligand (Bender et al., 2016). 1,000 models were generated. The top 200 models were selected using the highest DSASA values. From these top 200 models, the top 10 models were selected based on the lowest LigInterface values (Wisedchaisri et al., 2019). Visual analysis and images were generated using UCSF Chimera. Amino acids from Na_v_1.5 that are directly interacting with MEX binding site 1 or MEX binding site 2 were indicated using a 4.0 Å cutoff distance from the MEX molecules.

### Statistical Analysis

FitMaster (HEKA Elektronik, Lambrecht/Pfalz Germany), Excel (Microsoft, Seattle, WA), and GraphPad Prism5 (GraphPad Software, Inc., La Jolla, CA) were used for data acquisition and analysis. Data were presented as mean ± SEM. An unpaired Student’s *t* test, and one-way analysis of variance (ANOVA) followed by Newman-Keuls multiple comparison test and Kruskal-Wallis followed by Dunn’s *post hoc* test were used for the comparison of parametric and non-parametric data, respectively. *P* < 0.05 was considered statistically significant.

## Results

### Characterization of the LQT3 Patients

**Figure 1:**
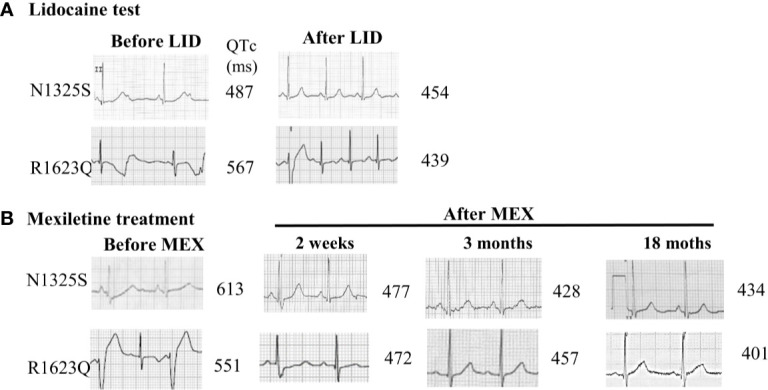
ECG records from lead II in LQT3 patients before and after lidocaine test and oral mexiletine treatment. **(A)** ECG records from lead II in LQT3 patients before treatment showed prolonged QTc interval and combined with a 2:1 atrioventricular conduction block (upper panel), the lidocaine test shortened the QTc interval. **(B)** After treatment with oral mexiletine, ECG showed a gradually shortening and normalized QTc interval in follow-up period.

A 15-year-old female was admitted because of recurrent transient loss of consciousness over 9 years. She has a family history of sudden cardiac death in three relatives, and another live relative had documented syncope but was not willing to accept any evaluation. The proband had been diagnosed with epilepsy during the past 5 years and was prescribed with lamotrigine and phenytoin, with reduced frequency of syncope from several times a year to one to two times a year. At presentation, her ECG showed remarkable prolonged corrected QT (QTc) intervals of 487–640 ms in sinus rhythm with ventricular rate of 50–72 beats per minute (bpm) and periodic atrioventricular (AV) block with 2:1 AV conduction (**Figure 1A**). A 24-h Holter monitoring documented 2:1 AV block with a ventricular rate of 42 bpm and 723 premature ventricular contractions (PVCs) with two morphologies and one episode of TdP. Her electroencephalogram was normal. Following intravenous administration of LID, the QTc interval was shortened from 487 to 454 ms (**Figure 1A**), and the 2:1 AV block disappeared. The patient was preliminarily diagnosed with congenital LQT3 based on the baseline ECG and the response to LID, and was treated with oral MEX alone at 200 mg daily. She refused implantable cardioverter defibrillator (ICD) therapy. This patient was followed up for 5 years with no episode of syncope and normal ECG recordings. Her QTc intervals in regular ECG and Holter monitoring remained between 477 to 434 ms (**Figure 1B**). AN1325S mutation of *SCN5A* gene, which was previously reported as a LQT3 causative mutation, was confirmed by molecular genetics.

A 6-year-old boy was admitted for frequent episodes of syncope in last 7 months, which often occurred at rest, and there was no family history of syncope or sudden cardiac death. The 24-h Holter monitoring documented a total of 69,388 ventricular ectopic beats with 55,067 single PVC, 1,880 paired PVCs, and 2,526 episodes of ventricular tachycardia (the longest episode of TdP lasted for 80.3 s). His ECG showed prolonged QTc intervals of 551–567 ms with frequent PVCs (**Figures 1A, B**). Following intravenous administration of LID, the QTc interval was shortened from 567 to 439 ms, and ventricular arrhythmias were decreased (**Figure 1A**). He was treated with oral MEX at 300 mg combined with 40 mg propranolol daily. During 2 years’ follow-up, no syncope occurred, and his QTc intervals showed a gradually shorter trend from 472 to 401 ms (**Figure 1B**). Furthermore, the 24-h Holter recordings after 1, 4, and 18 months showed 1,962, 18, and 0 PVCs, respectively, without any ventricular tachycardia. Genetics test indicated an R1623Q mutation of *SCN5A* gene, which was reported as a LQT3 causative mutation.

### Biophysical Properties of the WT and Mutant Channels

**Figure 2:**
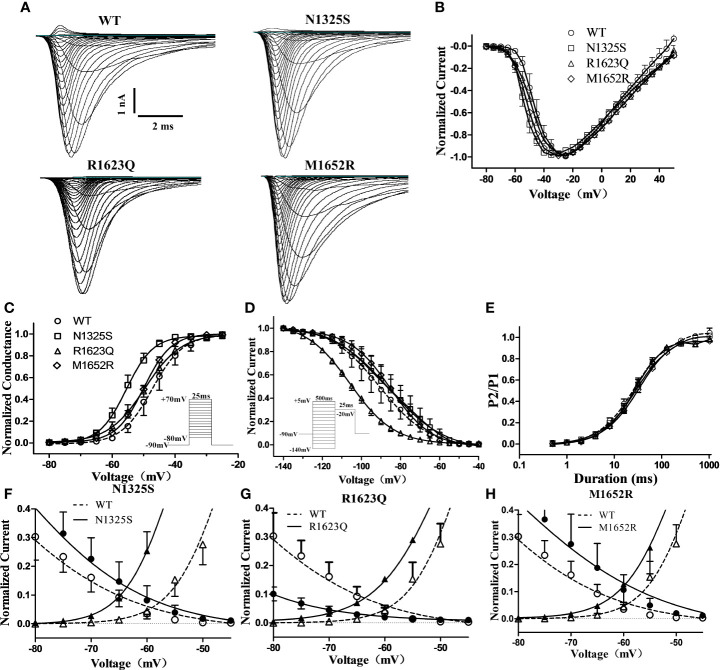
Electrophysiological characterization of WT and mutant SCN5A channels’ peak sodium current. **(A)** Represent traces of sodium current of WT and mutant channels. **(B)** Normalized I–V relationships of WT and mutant channels. Steady-state activation **(C)** and inactivation **(D)** curves of peak sodium current were determined with the inset protocol and fitted with a Boltzmann. **(E)** Time course of recovery from inactivation was fitted with a bi-exponential function. **(F**–**H)** Overlapping of activation and inactivation curve enlarged to show the window current of peak sodium current of mutant channels.

Though MEX may inhibit late *I*_Na_ in LQT3 patients carrying *SCN5A* mutations, some LQT3 patients with life-threatening arrhythmias were reported to be non-responsive to MEX, and the QTc intervals were not shortened by MEX in some mutations, including M1652R (Ruan et al., 2007). Here, the biophysical properties of three *SCN5A* mutations, i.e., N1325S and R1623Q (identified as MEX-sensitive mutations in the LQT3 patients in this study) and a previously reported MEX-insensitive missense mutation, M1652R, were investigated and compared. WT, N1325S, R1623Q, and M1652R mutant sodium channels were expressed in HEK293 cells, and their biophysical properties were measured by whole-cell patch-clamp techniques. Typical voltage-gated sodium currents were elicited in all channels and rapidly activated and inactivated by a series of depolarizing test potentials (**Figures 2A, B**). No significant differences in maximal peak sodium current density were observed among these channels. As shown in **Figure 2** and **Table 1**, there was a significant negative shift of steady-state inactivation curves of R1623Q mutant channel, i.e., more channels are inactivated at the holding potential of -90 mV, suggesting that R1623Q mutation may alter the expression or stability of Na_v_1.5 channel.

**Table 1:** Biophysical properties of mutant channels and inhibition of late *I_Na_* by drugs.

	Activation			Inactivation			Recovery			
	V_1/2_ (mV)	Slope, κ (mV)	n	V_1/2_ (mV)	Slope, κ (mV)	n	A1	τ_fast_ (ms)	τ_slow_ (ms)	n
WT	-47.3 ± 1.0	4.8 ± 0.8	7	-90.7 ± 1.8	-12.9 ± 1.8	6	0.32	24.0	164.0	7
N1325S(control)	-55.2 ± 0.5^*^	4.1 ± 0.6	13	-86.1 ± 1.3	-14.3 ± 2.8	13	0.35	23.6	170.0	13
N1325S(MEX)	-55.3 ± 0.5	4.9 ± 0.4	6	-95.62 ± 0.5^†^	-10.6 ± 0.5	5	0.09	67.2^†^	762.2^†^	6
R1623Q(control)	-49.6 ± 0.6	5.8 ± 0.6	7	-105.9 ± 0.7^*^	-11.3 ± 0.6	6	0.16	35.3	2356.3	9
R1623Q(MEX)	-51.7 ± 0.7	6.3 ± 0.7	6	-110.0 ± 0.8^†^	-11.0 ± 0.7	6	0.16	52.3	5007.5^†^	7
M1652R(control)	-49.9 ± 0.8	4.4 ± 0.3	6	-85.1 ± 1.0^*^	-14.4 ± 2.2	6	0.12	37.7	370.8	8
M1652R(MEX)	-52.2 ± 0.7	5.6 ± 0.6	6	-91.4 ± 2.0^†^	-16.0 ± 2.2	6	0.16	49.1	904.2^†^	8

*P < 0.05 compared with WT; ^†^P < 0.05 compared with control.

In contrast, compared to the WT channel, the steady-state activation curve of the N1325S channel was shifted by 7.9 mV toward more negative potentials (**Figure 2C** and **Table 1**), suggesting that N1325S mutant channels open at more negative membrane potentials, resulting in increased availability of the channels (Olesen et al., 2012). No significant differences in steady-state activation were observed between the WT and R1623Q or M1652R channels (**Figure 2C** and **Table 1**). However, both R1623Q and M1652R changed the steady-state inactivation curves of the channel. The R1623Q caused a negative shift of steady-state inactivation curves by 15.2 mV compared with WT channel. In contrast, the steady-state inactivation curves of the M1652R channels were positively shifted by 5.6 mV relative to the WT channel. No significant change was observed between the WT and N1325S channel in the steady-state inactivation (**Figure 2D** and **Table 1**).

The time course of recovery from inactivation was investigated by a two-pulse protocol. A 500 ms conditioning pulse (-20 mV) was used to induce inactivation followed by a test pulse (-20 mV) after returning to -90 mV for a variable interval to allow channels to transit from the inactivated state. There were no significant differences in the recovery kinetics among the four different channels (**Figure 2E** and **Table 1**). Finally, the window current of peak sodium current, resulting from the overlap of the activation and inactivation curves, was larger in all three mutant channels than that in WT (P < 0.05, **Figures 2F–H**).

**Figure 3:**
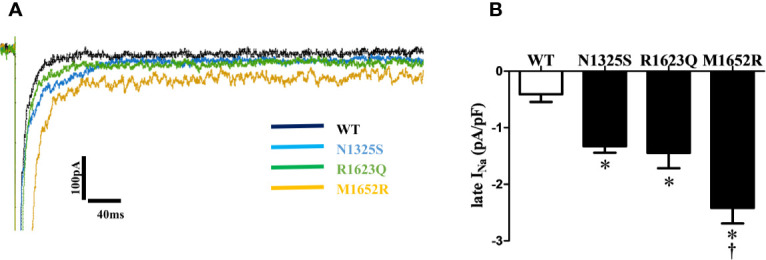
N1325S, R1623Q, and M1652R mutations increased late *I*_Na_. **(A)** Representative traces of late *I*_Na_ in WT, N1325S, R1623Q and M1652R mutant channels. **(B)** Bar graph summarized late *I*_Na_ at 200 ms, and showed that all three mutations increased late *I*_Na_ but with the greatest amplitude of late *I*_Na_ in the M1652R channel. Late *I_Na_* was measured at 200 ms after a voltage step from -90 to -20 mV. *P < 0.05, compared with WT; ^†^ *P* < 0.05, M1652R value versus N1325S and R1623Q.

Since late *I*_Na_ plays a critical role in LQT syndrome, we investigated the amplitude of late *I*_Na_ in these three mutant channels. Compared with the WT channel, all three mutations, N1325S, R1623Q, and M1652R channels, displayed a significantly increased late *I*_Na_ measured at 200 ms after depolarizing to -20 mV, with the greatest amplitude of late *I*_Na_ in the M1652R channel (**Figure 3**, -0.41 ± 0.14, -1.33 ± 0.11, -1.44 ± 0.27, and -2.23 ± 0.31 pA/pF, respectively, for WT, N1325S, R1623Q, and M1652R. n = 13, 15, 7, and 9. P < 0.05 vs. WT and M1652R, respectively).

### Electrophysiological Effects of MEX on Mutant Channels

**Figure 4:**
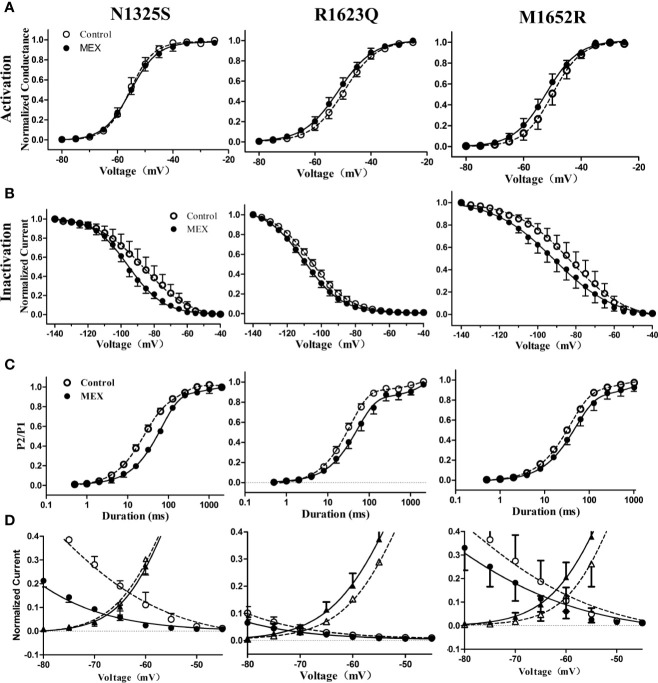
Effects of mexiletine (MEX) on the activation, inactivation, and recovery of mutant channels’ peak sodium current. Voltage dependence of activation **(A)** and inactivation **(B)** for mutant sodium channels in the absence and presence of MEX (10 μM). **(C)** Time course of recovery from inactivation under control and MEX was fitted using a bi-exponential function. **(D)** Window currents of peak sodium current in the absence (open symbols) or presence (filled symbols) of MEX (10 μM).

The effects of MEX on electrophysiological properties of N1325S, R1623Q, and M1652R channels were determined at a clinically relevant concentration of 10 µM (Wang et al., 1997). Though the steady-state activation of N1325S, R1623Q, and M1652R channels was not significantly affected by MEX (**Figure 4A** and **Table 1**), MEX caused a significant hyperpolarizing shift of steady-state inactivation curves in N1325S, R1623Q, and M1652R mutant channels by 11.6, 4.1, and 6.3 mV, respectively (P < 0.05, **Figure 4B** and **Table 1**). In addition, MEX delayed recovery process (**Figure 4C**) and reduced peak sodium’s window currents of all three mutant channels (**Figure 4D**). Thus, these effects could collaborate to reduce the availability of sodium channels

**Figure 5:**
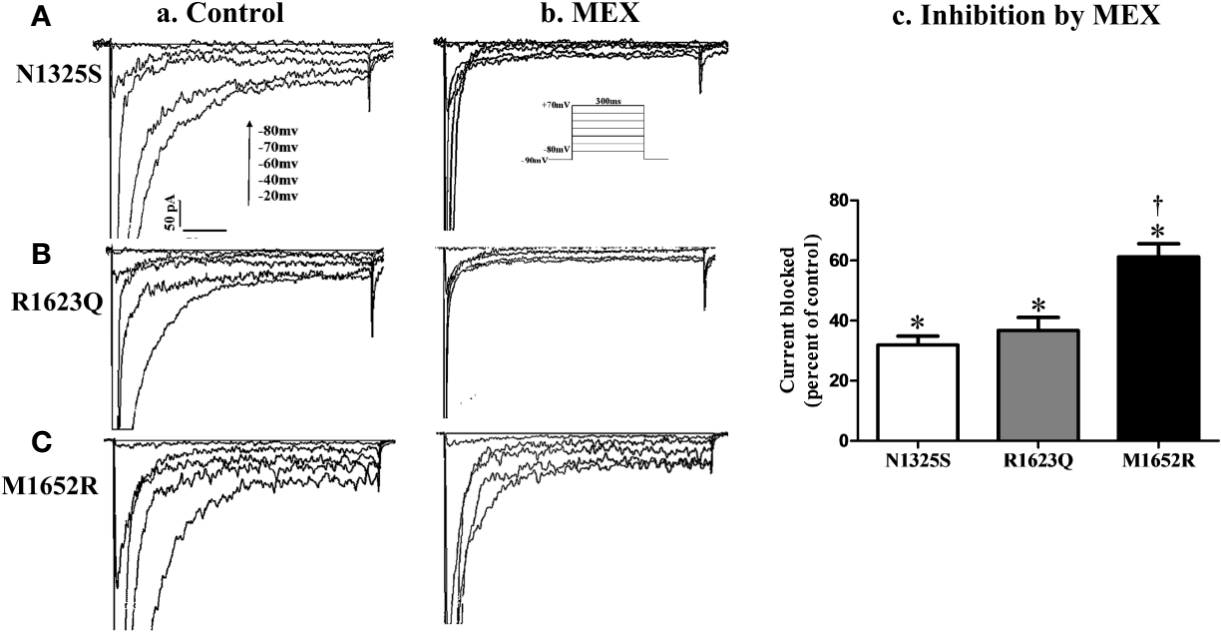
Effects of mexiletine (MEX) on late *I_Na_* of mutant channels. Representative traces of late *I*_Na_ recorded in the absence **(a)** or presence **(b)** of MEX (10 μM) at various depolarization voltages for N1325S **(A)**, R1623Q **(B)**, and M1652R **(C)** mutant channels, respectively. Summarized data of the percentage block of late *I*_Na_ by MEX was presented in panel **(c)**. Late *I*_Na_ was recorded at 10 min after MEX application and measured at 200 ms after a voltage step from -90 to -20 mV. Control values (no MEX) of late *I*_Na_ for N1325S, R1623Q, and M1652R were -1.33 ± 0.13, -1.22 ± 0.20, and -2.06 ± 0.21 pA/pF, respectively. N = 5–9. ^*^ *P* < 0.05 compared with control; *^†^P* < 0.05 compared with N1325S and R1623Q.

The effects of MEX on late *I*_Na_ were further investigated. Shown in **Figure 5** are the representative records of *I*_Na_ (primarily late *I*_Na_, a and b) and summarized data of late *I*_Na_ before and after MEX (n = 5–9, c) in N1325S, R1623Q and M1652R mutant channels (A, B, and C). Although MEX had differential clinical efficacy in patients with these mutations, MEX (10 µM) inhibited late *I*_Na_ in all three mutations. Furthermore, MEX exhibited a stronger inhibitory effect on the late *I*_Na_ of N1325S and R1623Q (by 68.12 ± 2.94% and 63.28 ± 4.29%, respectively) than that of M1652R mutant channels (by 38.82 ± 6.38%, *P* < 0.05 compared to that of N1325S and R1623Q). These results are consistent with the clinical response that MEX exhibited diminished effects on the MEX-insensitive mutant, M1652R, compared with the other two mutations.

**Figure 6:**
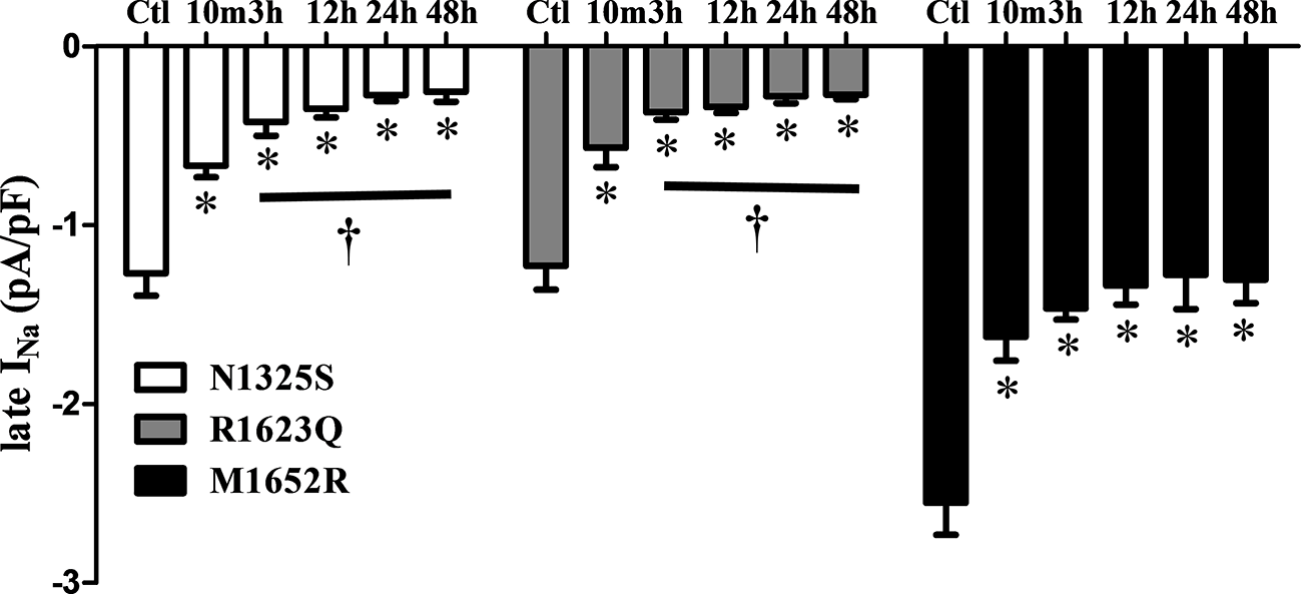
Mexiletine (MEX) inhibited late *I*_Na_ of N1325S and R1623Q mutant channels in a time-dependent manner. Late *I*_Na_ was measured from the cells with three individual mutations either without MEX treatment (control) or after incubation with 10 µM MEX for 10 min, and 3, 12, 24, and 48 h, respectively. MEX (10 μM) suppressed late *I*_Na_ of all three mutant channels after incubated with MEX for 10 min. The inhibition effects were more pronounced in N1325S and R1623Q mutant channels than that in M1652R mutation with the increases in incubation time. N = 5–9. ^*^ *P* < 0.05 compared with control; ^†^ *P* < 0.05 compared with 10 min’ exposure of MEX.

A gradually shortened QTc interval and reduced number of PVCs were observed over time with MEX treatment, esp. in the severe case of R1623Q mutation. Late *I*_Na_ in cells expressing the three mutant channels was measured after incubation with MEX (10 µM) for 10 min, and 3-, 12-, 24-, and 48-h. Compared to the control (no MEX**),** MEX (10 µM) suppressed the augmented late *I*_Na_ of all mutant channels after incubated with MEX for 10 min or longer (n = 5–9, **Figure 6**). Furthermore, the inhibitory effects were greater in N1325S and R1623Q mutant channels than in M1652R mutation with longer incubation time. Thus, MEX inhibited late *I*_Na_ in a time-dependent manner, suggesting that with longer incubation time, the inhibitory effect increased in MEX-sensitive mutations.

### Structural Modeling of MEX Binding to Mutant Na_v_1.5

**Figure 7:**
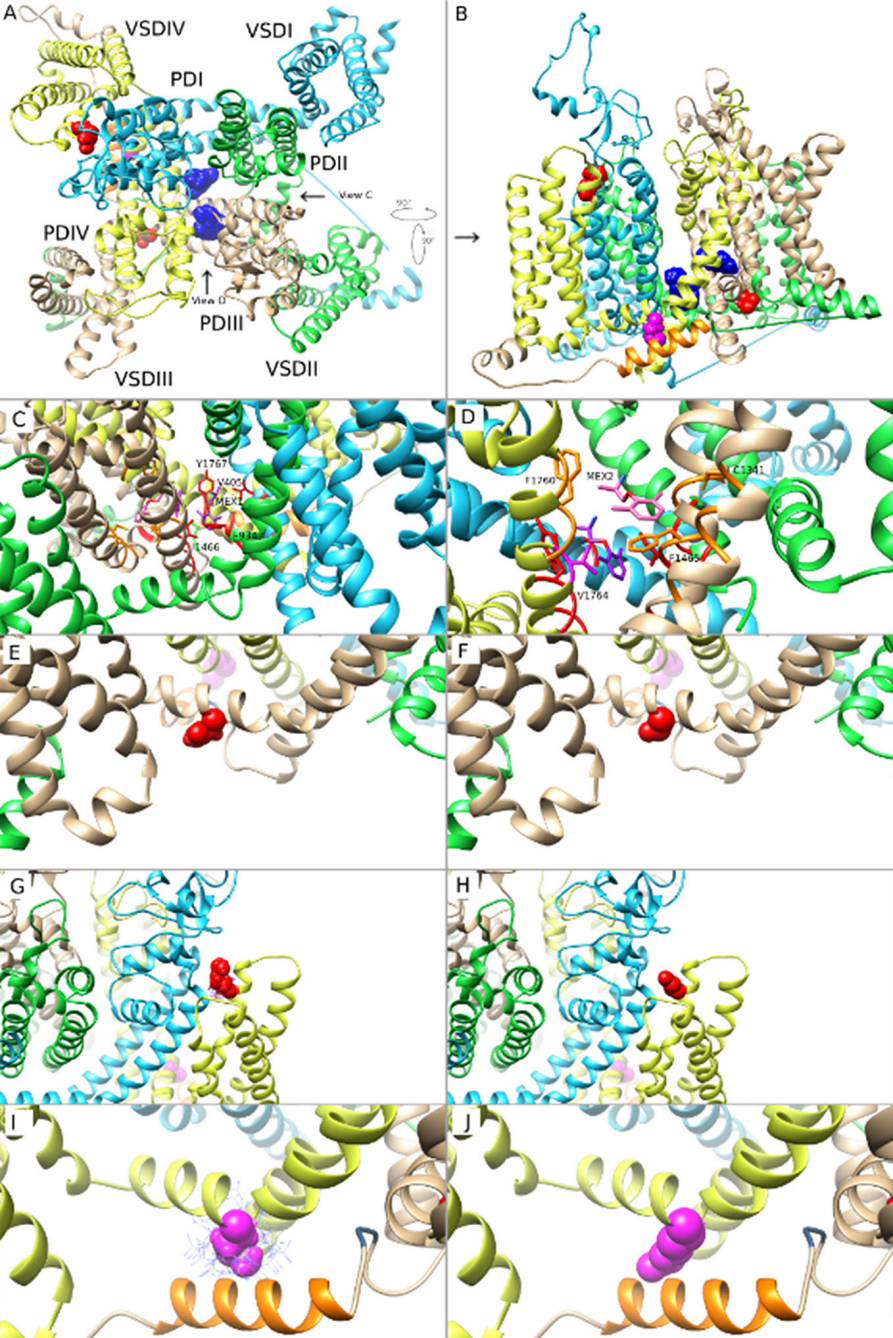
Molecular modeling of mexiletine (MEX) binding to MEX-sensitive (N1325S, R1623Q) and -insensitive (M1652R) mutant channels. **(A)** A molecular model of wild-type (WT) Na_v_1.5 in inactivated state, viewed from the extracellular side of the channel with MEX bound at two possible positions. **(B)** Side view of the channel with the three amino acids of interest facing the reader for clarity. **(C)** Closer view of MEX binding site 1. A single amino acid side chain in each binding region (S6 segments from all 4 domains) are labeled and colored. **(D)** Closer view of MEX binding site 2. Labels are specific to MEX binding site 2. A single amino acid side chain in each binding region (DIII-S5, DIII-S6, and DIV-S6) is labeled and shown. **(E**, **G**, **I)** Closer view of the three amino acid residues N1325, R1623, and M1652, respectively. **(F**, **H**, **J)**. Closer view of the optimal orientation for N1325S, R1623Q, and M1652R substitutions, respectively. Blue dots in panels **(I, J)** represent possible orientations of the M1652R substitution.

Structural modeling of MEX binding to Na_v_1.5 provided important molecular insights into how MEX interacts with the channel. The top 10 energetically favorable MEX - Na_v_1.5 models were analyzed revealing two possible binding sites within the Na_v_ channel pore. **Figures 7A, B** show both of the MEX binding sites occupied, with each site predicted by five models of the top 10 MEX - Na_v_1.5 models, making both sites equally possible. MEX is depicted in dark blue space filling model to illustrate the size of MEX molecule when bound to the channel. For orientation purposes, MEX binding site 1 is located in **Figure 7A** above MEX binding site 2. Amino acid residues (N1325 and R1623) are shown in red, and amino acid residue (M1652) is shown in magenta. Domain (D)I, DII, DIII, and DIV are shown as ribbons in cyan, green, brown, and yellow, respectively. The voltage-sensing domain (VSD) and pore-forming domain (PD) regions of each domain are labeled. Currently, there are no high-resolution structures of an open and conductive state of Na_v_1.5 channel; therefore, MEX binding to Na_v_1.5 was based on a partially open and presumably inactivated state of Na_v_1.5 channel (Nguyen et al., 2019).

The two MEX binding sites were formed by residues within the PD. **Figures 7C, D** shows a closer view of MEX binding site 1 and 2. Amino acid residues that directly contact binding site 1, 2, or both are shown in red, orange, and magenta, respectively (note that the amino acids colored in panels A and B are not the same as in panels C and D). MEX 1 and 2 molecules are shown in purple and pink, respectively while the heteroatoms on MEX 1, MEX 2, and amino acid cysteine are colored as follows: O = red, N = blue, S = yellow. The three mutations discussed in this paper are out of frame. MEX binding site 1 is formed by the S6 segments from all four domains (D) of the channel. The residues that directly contacted MEX include V405 and I408 (DI-S6), F934, L935, and L938 (DII-S6); I1466 and I1470 (DIII-S6); and V1764, Y1767, I1768, and I1771 (DIV-S6) (**Figure 7C**). MEX binding site 2 was located within the fenestration between DIII and DIV and formed by I1334, V1337, L1338, and C1341 (DIII-S5); T1461, L1462, and F1465 (DIII-S6); I1751, F1760, L1761, and V1764 (DIV-S6) (**Figure 7D**). We observed no evidence of two MEX molecules clashing with each other, despite a single residue (V1764) that binds both MEX molecules. Therefore, it is possible that both sites could be simultaneously occupied by two MEX molecules (**Figures 7C, D**).

Based on predicted MEX binding sites in our MEX - Na_v_1.5 models, there were no direct interactions between MEX and the three LQT3 mutated amino acid residues (N1325S, R1623Q, and M1652R). The findings suggested that these mutations altered the sensitivity of Na_v_1.5 to MEX through allosteric mechanisms by changing the conformation of Na_v_1.5 to become more or less favorable for MEX binding. N1325S in S4-S5 linker in DIII resulted in the substitution from large to smaller side chain. In addition, the side chain of N1325 residue is exposed to solvent in the inactivated state of Na_v_1.5 channel. Substitutions of side chains that are exposed to solvent are not likely to significantly impact the overall structure of the channel. **Figure 7E** shows the wild type N1325 residue, while **Figure 7F** shows the optimal position of the N1325S substitution. There is no evidence that the inactivated state of N1325S Na_v_1.5 channel would be disrupted (**Figures 7E, F**).

R1623 is the first arginine in the S4 segment in DIV VSD of Na_v_1.5. The model suggests that the R1623Q substitution, a smaller and polar amino acid, might affect the S4 movement during channel gating and therefore affect inactivated state of Na_v_1.5 (**Figures 7G, H**). The M1652 residue (in S4-S5 linker in DIV) has direct interactions with the α-helix immediately downstream from the isoleucine-phenylalanine-methionine (IFM) motif, the intracellular linker between DIII and DIV, which contributed to the fast inactivation gating mechanism of the channel. Specifically, M1652 was seen to interact with an aromatic amino acid Y1495 from the α-helix. This interaction is very favorable and allows for π stacking between the sulfur atom in M1652 and the aromatic ring of Y1495. Substitution of methionine to arginine (M1652R) resulted in the loss of this interaction. In addition, M1652R substitution is predicted to be disruptive as arginine has a much larger side chain that exhibits steric hindrance with residues Y1495 to K1500 in the α-helix (**Figures 7I, J**). This could be observed by the cloud of possible rotamer positions of the arginine side chain, whereby in the optimal position, it is very close to the α-helix backbone. The model suggests that M1652R mutation would be disruptive of the inactivated state and results in MEX-insensitive mutant Na_v_1.5 channel. These findings suggest that, from the perspective of the channel structure, mutations exhibit variable effects on the inactivated state of Na_v_1.5 channels, thus resulting in various affinity of MEX to sodium channels.

### Distinct Drug Responses of Mutant Channels

**Figure 8:**
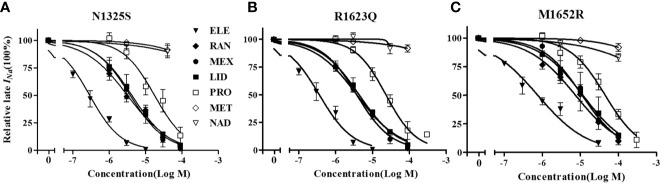
The concentration-response relationships of drugs on late *I_Na_* of mutant channels. **A-C**: Percentage inhibition of late INa in N1325S **(A)**, R1623Q **(B)** and M1652R **(C)** mutant channels in absence (0) and presence of increased concentrations of drugs. Late *I_Na_* was measured at 200 ms after a voltage step from -90 mV to -20 mV. ELE, eleclazine; RAN, ranolazine; MEX, mexiletine; LID, lidocaine; PRO, propranolol; MET, metoprolol; NAD, nadolol.

RAN and ELE, novel sodium channel inhibitors that exhibit greater potency and selectivity than class Ib antiarrhythmic agents for late *I*_Na_, have been shown to shorten the QTc interval in animal models and in patients with LQT3 (Chorin et al., 2016; Rajamani et al., 2016). Herein, we chose LID, MEX, RAN and ELE, as well as β blockers, propranolol, metoprolol, nadolol, to investigate their effects on these three mutant channels. As shown in **Figure 8** and **Table 2**, MEX, LID, RAN, ELE, and the β blocker propranolol, but not metoprolol and nadolol, significantly inhibited late *I*_Na_ *in vitro*. Inhibitory potency for late *I*_Na_ was quantified as the IC_50_ values calculated from the dose-response curves. Compared with the N1325S or R1623Q channel, the M1652R channel was much less sensitive to late *I*_Na_ inhibitors with greater IC_50_ values for MEX, LID, ELE, RAN, and propranolol.

**Table 2:** Inhibition of late *I_Na_* by drugs in mutant channels.

Drugs	N1325S		R1623Q		M1652R	
	IC_50_ (μM)	κ	IC_50_ (μM)	κ	IC_50_(μM)	κ
MEX	3.77 ± 0.33	0.90 ± 0.07	3.27 ± 0.30	0.93 ± 0.06	11.14 ± 1.18^*^	0.84 ± 0.08
LID	4.14 ± 0.53	0.90 ± 0.11	4.32 ± 0.39	0.82 ± 0.06	11.66 ± 2.82^*^	0.74 ± 0.18
ELE	0.27 ± 0.05	0.92 ± 0.09	0.34 ± 0.04	0.89 ± 0.11	0.74 ± 0.12^*^	0.61 ± 0.08
RAN	3.14 ± 0.36	0.73 ± 0.08	3.73 ± 0.36	0.74 ± 0.07	7.47 ± 0.75^*^	0.69 ± 0.05
PRO	17.60 ± 1.81	1.00 ± 0.12	23.11 ± 2.35	1.06 ± 0.14	45.04 ± 5.69^*^	0.93 ± 0.11
MET	~11.56 mM	0.60 ± 0.64	~20.98 mM	0.44 ± 0.33	~4.20 mM	0.65 ± 0.49
NAD	~2.03 mM	0.38 ± 0.32	~0.51 mM	0.89 ± 0.44	~3.80 mM	0.43 ± 0.17

*P < 0.05 compared with N1325S and R1623Q.

## Discussion

In the present study, two severe cases of LQT3 were successfully treated with MEX and mechanisms underlying the phenotype and responsiveness to MEX were investigated. The prolonged QTc interval, recurrent syncope, and documented episode of TdP in the young girl with N1325S mutation in *SCN5A* were reversed completely after MEX administration. Lamotrigine and phenytoin were partially effective, probably due to their inhibitory effects on *I*_Na_, specifically late I_Na_ (Chavez et al., 2015). LQTS is likely to be misdiagnosed with epilepsy because of the presentation of seizures, caused by cerebral hypoperfusion during ventricular arrhythmias. Indeed, mutations of *KCNQ1*, *KCNE1*, and *SCN5A* could also be found in certain epilepsy patients (Lupoglazoff et al., 2001). The AV block with 2:1 AV conduction block has been reported in patients with LQT2, LQT3, and LQT8 with an incidence of 4% in pediatric series and a greater than 50% probability of lethal arrhythmias within 6 months, regardless of the treatment (Lupoglazoff et al., 2001). The 2:1 AV conduction is likely due to the dramatic prolongation of ventricular repolarization, especially in His-Purkinje system, resulting in P wave falling on or before the preceding T wave, i.e. the effective refractive period in the AV junctional or His-Purkinje system (Rosenbaum and Acunzo, 1991; Pruvot et al., 1999). Hence, after shortening the QT interval with either LID or MEX, the functional AV block was reversed, as occurred in this patient. The boy harboring R1623Q mutation of *SCN5A* presented with not only extremely prolonged QTc interval but also large number of PVCs and episodes of TdP.

### Utility of Intravenous Infusion of *I*_Na_ Blockers in Different LQT Syndrome Subtypes

Intravenous infusion of *I*_Na_ blockers, if available, may be a quick tool to differentiate patients with LQT3, and to predict the efficacy of long-term treatment with *I*_Na_ blockers. Moritoshi et al. reported that MEX induced greater QTc shortening in LQT3 patients than in LQT1 and LQT2 patients and concluded that MEX infusion test is a useful tool to distinguish LQT3 from LQT1 or LQT2 (Funasako et al., 2016). LID test was reported to be useful in distinguishing pathogenic LQT3 mutations from other *SCN5A* variants of uncertain significance (Anderson et al., 2017). Our study demonstrates that QTc interval was remarkably shortened and the arrhythmic activities were abolished with the intravenous injection of LID, suggesting that LID test may be useful in determining the efficacy of MEX and to provide a rapid control of TdP in severe cases. Considering the similarity between LID and MEX (Dumaine et al., 1996), these two patients were treated with oral MEX, which shortened the QTc interval to normal range and abolished ventricular arrhythmias and syncope associated with QT prolongation without the need of ICD implantation. Additionally, the QTc interval and PVCs of the patient with R1623Q mutation reduced gradually over the treatment period. It may suggest that MEX has an accumulation effect on late *I*_Na_, possibly due to remodeling of the late *I*_Na_, as shown in the two patients. Finally, future studies are required to further support that the use of LID test in identifying MEX-sensitive mutations in LQT3 patients.

### Gating Changes in LQT3 Mutations

N1325S mutation in *SCN5A* is one of the earliest mutations reported to be associated with LQTS (Wang et al., 1995), in which asparagine is substituted by serine at position 1325 in the DIII/S4-S5 intracellular linker of Na_v_1.5. The R1623Q mutation is located in the S4 segment in DIV VSD of Na_v_1.5. The M1652R mutation, a MEX-insensitive mutation, is located in the DIV/S4-S5 intracellular linker of the channel. Similar to previous studies (Ruan et al., 2007), gating defects were demonstrated in all three mutations.

In the current study, the gating properties of N1325S, R1623Q, and M1652R channels were studied in HEK293 cells. Typical voltage-gated sodium currents were elicited in all channels, though no significant differences in maximal peak sodium current density were observed among these channels. For R1623Q mutant channel, more channels are inactivated at a physiological holding potential (-90 mV) compared to the other two mutant channels. Therefore, R1623Q mutation may alter the expression or stability of Na_v_1.5 channel. However, similar expression of WT and R1623Q channels was previously reported (Makita et al., 1998). Nonetheless, the use of heterologous expression system may not be the most suitable system to determine the expression, trafficking, and stability of the channels. Future studies in iPSC-derived cardiomyocytes or transgenic animals are needed to further interrogate for possible changes in the mutant channels.

A previous study has shown that MEX preferentially binds to the inactivated state of the sodium channel (Desaphy et al., 2001); therefore, mutations that favor the inactivated state may facilitate MEX binding to the channel with an increased clinical efficacy. Accordingly, MEX-sensitive mutation, R1623Q, causes a hyperpolarizing shift of the steady-state inactivation curve, which would favor the presence of the sodium channel in the inactivated state. In contrast, the MEX-insensitive mutation, M1652R, causes a depolarizing shift of steady-state inactivation curve. Therefore, our study supports the notion that the inactivated state of sodium channel may be an important factor that determines MEX sensitivity and the clinical response of MEX treatment. Indeed, this is consistent with the previous report (Ruan et al., 2007; (Moreno et al., 2019). The N1325S mutation, another MEX-sensitive mutation, causes a hyperpolarizing shift in the steady-state activation curve, increasing the activation of the channel.

### Molecular Insights Into MEX Sensitivity

Molecular modeling of N1325S, R1623Q and M1652R mutations provides structural insights into possible mechanisms for how these mutations may alter the sensitivity of the Na_v_1.5 channel to MEX. In addition, it also emphasizes the critical importance of future studies to resolve an open structure of human Na_v_ channel. Consistent with the electrophysiological findings, the molecular modeling suggests that the M1652R mutation affects the inactivated state of the channel (**Figure 7**). Based on the computational modeling, one possible mechanism of how M1652R results in MEX-insensitive channel is by not allowing the channel to fully inactivate and thus increasing the transition time from open to inactivated state. Mutation of R1623Q from a large basic amino acid to a smaller polar amino acid may affects the S4 movement during channel activation. The findings are consistent with our patch-clamp analyses where the R1623Q resulted in a hyperpolarizing shift of the steady-state inactivation curve and thus favoring the inactivated state. N1325S mutation may not significantly disrupt the inactivated state and the transition time from open to inactivated state is predicted to be faster. Thus, these two mutant channels favor inactivation state of channels and remain sensitive to MEX. However, Na_v_1.5 structure in an open state channel is needed to provide further support. In addition, future studies are needed to reveal conformational changes of the channel between the drug-free and drug-bound states.

One previous report (Ruan et al., 2007) also reported that inactivated state of sodium channel favors inhibition by MEX, however, the peak, but not late *I*_Na_ was studied. Since the late *I*_Na_ plays critical roles in LQT3, we investigated the relationship of late *I*_Na_ and the patient’s sensitivity to MEX. Multiple mechanisms may result in an increase in late *I*_Na_. Single-channel records have revealed that channel bursting and late reopening are responsible for the generation of late *I*_Na_ in various mutations (Bennett et al., 1995; Dumaine et al., 1996). An additional mechanism for a sustained late current is the overlap between the channel activation and inactivation (Wang et al., 1996), resulting in a fraction of channels remaining open. Other mechanisms that may contribute to the late current include non-equilibrium gating processes causing channel re-opening due to more rapid recoveries from inactivation (Chadda et al., 2017). In our study, we found that all three mutations increased window currents of peak *I*_Na_ and late *I*_Na_ compared with the WT channel, with the largest late *I*_Na_ in M1652R compared to the two MEX-sensitive mutations (**Figure 3**). In view of the critical role of late *I*_Na_ in LQT3, this effect may account for the patients’ phenotypes. There is no sign of mechanism of changes of channel re-opening, and late *I*_Na_ generated at -20 mV is out of voltage “window”, so the augmented late *I_Na_* are probably due to slower inactivation kinetics.

MEX has been shown to be effective in suppressing malignant ventricular arrhythmias and reducing the risk of sudden cardiac death in LQT3 patients and animal models. In addition, the prolonged APD and late *I*_Na_ of N1325S transgenic mice could be reversed by MEX (Tian et al., 2004). Although these three mutations showed distinct clinical responses to MEX, our results demonstrate that MEX has similar effects on all three mutations *in vitro*, including significant hyperpolarizing shift of steady-state inactivation curves, reduced window currents, and delayed recovery of channels into activated state (**Figure 4**). In accordance with these results, the augmented late *I*_Na_ in all three mutant channels was suppressed by MEX. Therefore, it can be concluded that MEX could suppressed late *I*_Na_ by stabilizing the inactivated state of the channel as evidenced by a hyperpolarizing shift in the steady-state inactivation curve and the slow recovery from inactivation.

A gradually shortened QT interval and reduced PVCs with treatment time for MEX-sensitive mutations, indicating that MEX might have an accumulation effect on late *I*_Na_. MEX could suppress late *I*_Na_ of all three mutant channels after incubated with MEX for 10 min, but the inhibitory effects were more pronounced in N1325S and R1623Q mutant channels with longer incubation time. MEX may penetrant into the cell in a slow fashion through the membrane, possibly leading to the required longer incubation time and slower action in inhibiting late *I*_Na_.

Effects of beta-blockers on LQT3 are controversial as they decrease the heart rate, which may result in the augmentation of late *I*_Na_ (Wu et al., 2011). Propranolol blocks *I*_Na_ in a manner similar to local anesthetic drugs, which may contribute to its anti-arrhythmic effects in LQT3 patients (Bankston and Kass, 2010). In this study, we tested the effects of several clinical relevant medicines on late *I*_Na_ *in vitro*. As shown in **Figure 8** and **Table 2**, propranolol, but not metoprolol or nadolol, inhibited late *I*_Na_. Therefore, if second drug is needed, non-selective beta-blockers with inhibitory effects on late *I*_Na_, such as propranolol, rather than metoprolol and nadolol, would be preferred in patients with LQT 3.

The drug responses of different LQT3-causative mutations and the use-dependent block of peak *I*_Na_ was associated with the clinical efficacy of MEX in LQT3 (Ruan et al., 2007). As shown in **Table 2**, sodium channel blockers (LID, MEX, ELE, and RAN) and propranolol suppressed late *I*_Na_, with ELE having the highest potency. In addition, the M1652R mutant channel showed reduced responsiveness to *I*_Na_ blockers and propranolol with IC_50s_ being about three times greater than that of N1325S and R1623Q. Though the patient with M1652R responded poorly to MEX treatment, MEX inhibited late I_Na_ of M1652R mutant channel *in vitro*. The IC_50_ of M1652R is ~3–4 fold higher than that of the other two mutations. In addition, M1652R exhibits greater amplitude of late I_Na_ with larger residual late I_Na_ after MEX application compared to N1325S and R1623Q. All these effects may account for the less clinical efficacy in patients harboring M1652R mutation. Thus, these findings suggested that drug screening in cells expressing disease-causing mutant channels would be helpful to predict the efficacy of drugs in LQT3 patients. Selective late *I*_Na_ blockers with a greater potency in inhibiting late *I*_Na_, such as ELE, may be effective in patients with MEX-insensitive mutations, e.g., M1652R.

Na^+^ channel inhibitors are supposed to bind to a local anesthetic site of Phe1760 and Tyr1767 in Nav1.5 (Kass and Moss, 2006). Mutations located in close to this binding site such as F1760A/Y1767A would disrupt the binding of drugs such as MEX, ELE, and propranolol (Sasaki et al., 2004; El-Bizri et al., 2018). The gating state of sodium channels also plays an important role in drug interaction (Desaphy et al., 2001). Furthermore, besides the gating properties, other factors such as therapeutic adherence, pharmacokinetic and metabolic factors of drugs, interaction among cardiac ion channels, and other modulators are also important determinants for the response to therapy. These factors may alone or in combination with each other contribute to the variable response to drug therapy. Development of more potent late *I_Na_* inhibitors may be beneficial to patients with MEX insensitive mutations. Further large-scale investigations on these factors and whether this kind of current-specific treatment could reduce the need or delivery of shocks for ICD implantation are needed.

## Conclusion

MEX suppresses late *I_Na_* of N1325S, R1623Q, and M1652R mutations with abnormal gating properties of mutant channels and stabilizes the inactivated state of the channels through a hyperpolarizing shift of the steady-state inactivation curve and a slowed recovery from inactivation with greater potencies in N13255S and R1623Q than that in M1652R, which is consistent with the clinical response to MEX in patients. LID test, molecular modeling, and screening for more potent drugs are useful for the treatment of patients with different mutations of LQT3.

## Data Availability Statement

The raw data supporting the conclusions of this article will be made available by the authors, without undue reservation, to any qualified researcher.

## Ethics Statement

The studies involving human participants were reviewed and approved by Ethics Committee of Peking University First Hospital. Written informed consent to participate in this study was provided by the participants’ legal guardian/next of kin. Written, informed consent was obtained from the minor(s)’ legal guardian for the publication of any potentially identifiable images or data included in this article.

## Author Contributions

GL, NC, and LW constructed the concept and designed the experimental protocol. GL, RW, C-YW, LR, and VY-Y performed experiments and molecular model. P-XH, S-DY, and X-QL collected clinical data. GL, RW, and DH analyzed data and interpreted results of experiments. GL and RW prepared figures and drafted this manuscript. GL, RW, LR, VY-Y, DH, NC, and LW edited and revised this manuscript. All authors contributed to the article and approved the submitted version.

## Funding

This work was supported in part by grants from the National Natural Science Foundation of China (81270253, 81670304, 81430098, 81170156, 81770325 and 81930105), NIH R01 HL085727, NIH R01 HL085844, and NIH R01 HL137228 (NC), VA Merit Review Grant I01 BX000576 and I01 CX001490 (NC), NIH R01 HL137228-S1 (RW) and American Heart Association Predoctoral Fellowship Award 18PRE34030199 (LR).

## Conflict of Interest

The authors declare that the research was conducted in the absence of any commercial or financial relationships that could be construed as a potential conflict of interest.
